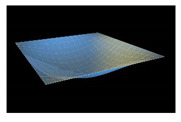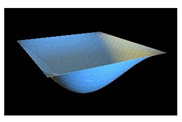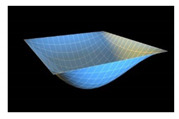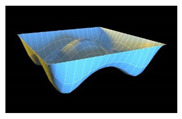# Stability Improvement of Flexible Shallow Shells Using Neutron Radiation

**DOI:** 10.3390/ma13143187

**Published:** 2020-07-16

**Authors:** Anton V. Krysko, Jan Awrejcewicz, Irina V. Papkova, Vadim A. Krysko

**Affiliations:** 1 Department of Mathematics and Modeling Saratov State Technical University, 77 Politehnicheskaya Str., 410054 Saratov, Russia; anton.krysko@gmail.com (A.V.K.); ikravzova@mail.ru (I.V.P.); tak@san.ru (V.A.K.); 2 Department of Automation, Biomechanics and Mechatronics, Lodz University of Technology, 1/15 Stefanowski St., 90-924 Lodz, Poland

**Keywords:** buckling, microstructures, stress relaxation, vibration

## Abstract

Microelectromechanical systems (MEMS) are increasingly playing a significant role in the aviation industry and space exploration. Moreover, there is a need to study the neutron radiation effect on the MEMS structural members and the MEMS devices reliability in general. Experiments with MEMS structural members showed changes in their operation after exposure to neutron radiation. In this study, the neutron irradiation effect on the flexible MEMS resonators’ stability in the form of shallow rectangular shells is investigated. The theory of flexible rectangular shallow shells under the influence of both neutron irradiation and temperature field is developed. It consists of three components. First, the theory of flexible rectangular shallow shells under neutron radiation in temperature field was considered based on the Kirchhoff hypothesis and energetic Hamilton principle. Second, the theory of plasticity relaxation and cyclic loading were taken into account. Third, the Birger method of variable parameters was employed. The derived mathematical model was solved using both the finite difference method and the Bubnov–Galerkin method of higher approximations. It was established based on a few numeric examples that the irradiation direction of the MEMS structural members significantly affects the magnitude and shape of the plastic deformations’ distribution, as well as the forces magnitude in the shell middle surface, although qualitatively with the same deflection the diagrams of the main investigated functions were similar.

## 1. Introduction

Since thin-walled shells are highly efficient structural members, they have wide practical application. For example, they are used in instrument making, aerospace, petrochemical and nuclear industries. In general, shell structural elements can be made of various materials: concrete, reinforced concrete (supporting structures of nuclear reactors), various alloys and composites, etc. The mentioned materials can be used for fabrication of microelectromechanical systems (MEMS) including electromechanical relays, force and pressure sensors, accelerometers, digital logic switches, as well as biosensors and micromirrors. The latter composite devices are typically exposed to temperature and neutron irradiation as well as external force action during their working regimes. Hence, the use of novel and challenging MEMS designs and structural elements in space exploration to maintain the integrity of sensors and ensure operation in harsh conditions may play an important role for future space explorations.

In particular, flexible rectangular shallow shells are more useful for serving as MEMS resonators. The introduction of MEMS for operation in adverse environmental conditions, such as exposure to neutron irradiation and temperature risks, requires the use of new MEMS designs and structural elements in order to maintain the integrity of sensors as well as to ensure operation under various conditions.

It should be emphasized that, if the requirement for strength and durability of materials is presented for structural elements in the macroscale, then for the microscale the change in the sensitivity and stable operation of the device under various input signal modes comes to the fore.

Already in 1953, Dienes [1] pointed out the importance of nuclear radiation with perspectives on changing the mechanical properties of solids. Not only did he show how the mechanical properties of crystalline substances—and in particular metals—can be produced by fast particle irradiation, but he also discussed how in high polymers nuclear radiation influenced the subsequent chemical reaction theory by altering their properties.

Further research on the analysis of stresses in metals caused by volumetric changes during neutron irradiation was carried out by Remnev [2,3]. Lensky [4] noted that a significant change in mechanical properties, considering the uneven volume distribution, requires clarification of existing and new theories as well as development of methods for calculating structural elements, especially if they are exposed to radiation fluxes during an operation. Ilyushin and Ogibalov [5] discussed the method of calculating the strength of thick-walled cylindrical shells in the presence of radiation. Norris [6] employed electron irradiation with the beam of a high voltage electron microscope to produce void growth in metals. Moreover, investigations of void growth effects were discussed for stainless steel and for nickel with emphasis on the moving dislocations and the surfaces. In the monograph by Likhachev and Pupko [7], the general theory relations of flow for irradiated materials were presented, based on the models proposed in the works of Birger for nonirradiated materials. Moreover, key generalizations for the governing equations of the plasticity and creep theory were made by taking into account irradiation with a neutron flux.

Singh et al. [8] studied the influence of irradiation with fission neutrons on specimens of pure copper and copper alloys. The change in volume due to irradiation as well as radiation damage of microstructures was reported.

Singh et al. [9] studied the features of neutron irradiation on microstructure and mechanical properties of pure iron by transmission electron microscopy. It was illustrated how interaction of cleared channels influenced the deformation and fracture behavior of the irradiated pure iron.

Garner et al. [10] studied swelling and creep phenomena irradiated in EBR-II. They illustrated how the swelling reached high levels rather quickly by reducing both fuel pin cladding interaction and the contribution of the irradiation to the total deformation.

Shea [11] reviewed the sensitivity of MEMS devices to radiation. The latter was linked primarily to the impact on device operation of radiation-induced trapped charge in dielectrics, with emphasis to electrostatic principles. It was illustrated how to increase radiation tolerance by design using either the actuation principle or electrical shielding of dielectrics.

Ghorbanpour Arani et al. [12] investigated the buckling of a cylindrical shell in the neutron radiation environment under combined static and periodic axial forces. They illustrated how the radiation induced porosity in elastic materials changed their thermal, electrical and mechanical properties. The carried out analysis was based on the classical shell theory. It was shown that both neutron induced swelling and temperature actions exhibited important effects on the stability and buckling of the cylindrical shell.

Sercombe et al. [13] used 3D simulation with an account of burnup-dependent pellet-clad friction while studied the power ramped cladding stresses during bare irradiation and ramp tests. They explained how the sole evolution of the friction coefficient with burnup captured the radial crack pattern of the rodlets after power ramping. Moreover, they derived a simple relation between the friction coefficient and the burnup variations.

Ben De Pauw [14] employed the optical fiber sensors to measure the fuel assembly vibration in a lead–bismuth eutectic cooled nuclear installation as input to assess vibration-related safety hazards.

Sitepu et al. [15] used palm fiber and palm shell as a thermal neutron radiation shielding material based on the neutron activation analysis and studied thermal neutron flux on gold pieces.

In studies [11,12,13,14,15,16,17], a generalized virtual modeling method using modern computer technologies to solve one-dimensional and two-dimensional nonlinear problems was proposed.

Srour et al. [18] carried out historical review of the literature regarding the radiation-induced displacement damage effects in semiconductor materials and devices. In particular, the effects of uniform radiation-induced displacement damage in bulk material and Si devices were outlined.

The increasing popularity of MEMS devices has led the researchers and engineers to study radiation-resistant resonators. Structural members of MEMS devices are microsized and very sensitive to external influences. They also depend on their respective operating principles. For example, for the oscillators used in GPS systems, one millionth of a percent is required for their operation stability, while the transistors used in integrated circuits can withstand parametric changes of several percent [18].

Babich [19] proposed mathematical models of radiation-induced physical processes in materials. Thermal and radiation strains dependent on the energy spectrum and components of radiation were estimated based on an appropriate analytical method. Moreover, the influence of the effects of radiations on the stress–strain state of thin plates was studied.

Marchal et al. [20] employed the finite element method to analyze the phenomenon of Pellet-Cladding interaction in nuclear fuel rods. The latter interaction produced high large stresses and led to failure. The fuel rods belong to the main members of nuclear water reactors and they consist of zirconium alloy tubes containing uranium dioxide pallets. The authors reported that the irradiation-induced phenomena yielded a densification of the fuel material in the irradiation early stages followed by solid swelling effects.

Semenov and Woo [21] modeled and analyzed dislocation structure development in metals and alloys with an account of anisotropic nucleation and dislocation loops subjected to actions of fast neutrons and external stresses. The stress-induced dislocation anisotropy was detected which exhibited a strong correlation with swelling. The nucleation kinematic was studied based on the derived Fokker-Planck equation.

Karahan and Buongiorno [22] formulated an engineering oriented code to predict the irradiation behavior of U–Zr and U–Pu–Zr metallic alloy and UO_2_–PuO_2_ mixed fuel pins in sodium-cooled fast reactors. Based on the introduced fuel pin geometry, composition and irradiation history, the authors studied numerically the fuel and clad thermomechanical behavior as both steady-state and design-basis transient scenarios.

Hall Jr. [23] derived the continuum plasticity model and studied the irradiation induced swelling of reactor core materials. It was shown that in the presence of significant swelling the deviatory and volumetric strain rate components are functions of both deviatory and hydrostatic components of stress for both linear and nonlinear creep.

Williamson [24] developed a powerful multidimensional fuels performance analysis having capability to simulate multi pellet steady and transient rod behavior based on the ABAQUS thermo mechanics code. Numerous input parameters were taken into account, including temperature, fuel densification, fission gas release during irradiation and gap heat transfer, etc.

The published experimental data presented in [18,19,20,21,22,23,24] show that, under the radioactive irradiation influence, a significant change is observed both in the thermo physical and elastic properties, as well as in the short-term and long-term mechanical characteristics of materials.

Karthik et al. [25] carried out the systematic microstructural studies of the austenitic stainless steel irradiation performance. The following salient features were analyzed: (a) behavior of SS316 at different fluence levels and (b) irradiation experiments of classical and modified D9 versions.

Fisher and Longo [26] carried out the creep analysis of slightly oval cylindrical shells under time-dependent loading, temperature and neutron flux. The influence of variations in neutron flux, external pressure, mesh size and solution time step on collapse time was investigated based on the derived equilibrium equations.

NASA’s Jet Propulsion Laboratory in Pasadena (JPL) developed micro and nano technologies for space exploration [27], while Sandia National Laboratory efforts to create radiation-strengthened inertial sensors [28]. The Defense Threat Reduction Agency (DTRA) of the United States has initiated several programs at several universities and government laboratories to study the radiation effects on a number of materials and structural elements of various MEMS and NEMS types. Investigations include silicon, silicon carbide, piezoelectric and ferroelectric, electro–optical and 2D materials, as well as studying the effect of radiation and thermal effects on the electrical, mechanical and optical properties and manifestations in various MEMS and NEMS operating modes.

In the last decade, exotic properties of pantographic metamaterials have been investigated and various mathematical models (both discrete and continuous) have been introduced.

It should be mentioned that the lack of studies devoted to stability of flexible rectangular shallow shells irradiated with neutrons may be partly due to the problem complexity, which is associated with the lack of an adequate mathematical model. At the same time, an experimental study of prototype structures both on a macro scale and on a micro scale is expensive and is associated with the destruction of test radioactive components. Therefore, there is a growing interest in using mathematical modeling methods to study various structural elements and our study aims at filling the existing gap in the so far described research.

The investigation of problem for the stability and stress–strain state of flexible shallow rectangular shells in terms of neutron irradiation is currently necessary both from a theoretical as well as practical point of view. This observation follows from a critical review of the works [29,30,31].

This work is devoted to the modeling and prediction problem of the flexible rectangular shallow shell stability subjected to neutron irradiation, used as MEMS resonators. The validity of our research can be also justified based on the observation that to the best of our knowledge, a study of the geometrically and physically nonlinear shells stability under the neutron irradiation action has not been done.

The study is organized in the following way. The shell mathematical model is introduced in Section 2, whereas the employed computational methods are presented in Section 3. The reliability and validity of the obtained results are discussed in Section 4. Section 5 deals with the account of the residual shell plastic deformations and the numeric examples are reported in Section 6. Section 7 presents the concluding remarks.

## 2. Problem Statement

**Figure 1:**
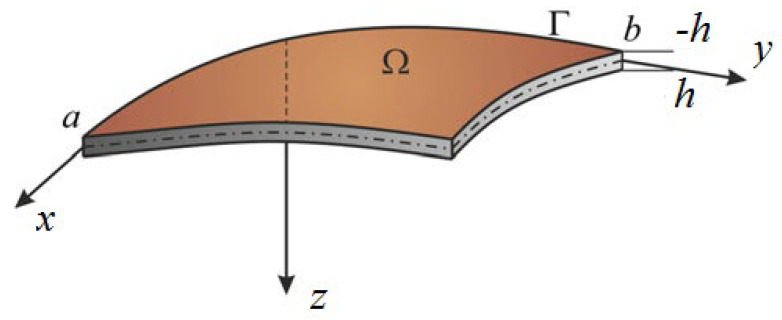
Computational shell scheme.

We consider a shell of length a, with thickness 2h and width b. It occupies the volume $\Omega =\left\{0\leq x\leq a;\;0\leq y\leq b;\;-h\leq z\leq h\right\}$, where *x*, *y*, *z* are spatial coordinates (Figure 1).

The following hypotheses hold:

(i) the Kirchhoff–Love kinematic shell model is taken where strain tensor components for the middle shell surface are $\varepsilon_{xx}=-z\frac{\partial^{2}w}{\partial x^{2}},\;\varepsilon_{yy}=-z\frac{\partial^{2}w}{\partial y^{2}}$ and *w*(*x*, *y*, *t*) denotes shell deflection at its middle surface;
(ii) the shell material is isotropic, but nonhomogenous and its properties depend on temperature, T(*x*, *y*, *z*), i.e., we have elastic modulus E and Poisson’s ratio *ν* are constants at the initial time moment and then the functions *E* = *E*(*x*, *y*, *z, e_r,_ T*(*x*, *y*, *z), α(T)*), *ν*= *ν*(*x*, *y*, *z, e_r,_ T*(*x*, *y*, *z), α(T)*) and the shell deformed state depends on temperature at a point (x, y, z);
(iii) Duhamel–Neumann law is adopted, i.e., we have $\varepsilon_{xx}^{z}=\varepsilon_{xx}+\varepsilon_{xx}^{1p}+\alpha T(x,y,z),\;\varepsilon_{yy}^{z}=\varepsilon_{yy}+\varepsilon_{yy}^{1p}+\alpha T(x,y,z),\;\varepsilon_{xy}^{z}=\varepsilon_{xy}+\varepsilon_{xy}^{1p},$ where α stands for coefficient of linear thermal extension and $\varepsilon_{xx}^{1p},\;\varepsilon_{yy}^{1p},\;\varepsilon_{xy}^{1p}$ are plastic components of the strain tensor components at the unloading time;
(iv) theory of plasticity and Mises criterion are used;
(v) deformation diagrams *σ_r_* (*e_r_*, *T*, *α*) depend both on shell temperature and its stress–strain state, where *σ_r_* stands for intensity of tensions and er is deformation intensity;
(vi) the temperature field is defined by a 3D heat-transfer equation without any restrictions put on temperature distribution.

The mathematical model is yielded by the Hamilton’s variational principle. The shell accumulated potential energy is:

(1) $$\prod =\frac{1}{2}\int_{-h}^{h}dz\int_{0}^{a}\int_{0}^{b}\left(\sigma_{xx}\varepsilon_{xx}+\sigma_{yy}\varepsilon_{yy}+2\sigma_{xy}\varepsilon_{xy}\right)dxdy,$$

where *σ_xx_*, *σ_yy_*, *σ_xy_*—stress tensor components for the shell middle surface.

Moreover, the works of external forces.

(2) $$\delta^{\prime }W=\int_{0}^{a}\int_{0}^{b}q\delta wdxdy.$$

where *q*—external transverse uniformly distributed load.

Stress–strain state of nonhomogenous plates and shallow rectangular shells is governed by the following nonlinear PDEs:

(3) $$\left(\begin{array}{cc} L_{11} & L_{12}\\ L_{21} & L_{22} \end{array}\right)\cdot \left(\begin{array}{c} w\\ F \end{array}\right)+\left(\begin{array}{cc} L & 0\\ 0 & -\frac{1}{2}L \end{array}\right)\cdot \left(\begin{array}{c} w,F\\ w,w \end{array}\right)=-\left(\begin{array}{c} Q_{1}\\ Q_{2} \end{array}\right),$$

where *F* is the load function and the linear operators with variable coefficients are defined in the following way:

(4) $$\begin{array}{l} L_{11}\left(•\right)=\frac{\partial^{2}}{\partial x^{2}}\left(\lambda^{-1}B_{11}^{0}\frac{\partial^{2}\left(\cdot \right)}{\partial x^{2}}+B_{10}^{*}\frac{\partial^{2}\left(\cdot \right)}{\partial y^{2}}\right)+\\ \;+2\frac{\partial^{2}}{\partial x\partial y}\left[\left(B_{11}^{*}-B_{10}^{*}\right)\frac{\partial^{2}\left(\cdot \right)}{\partial x\partial y}\right]+\frac{\partial^{2}}{\partial y^{2}}\left(B_{10}^{*}\frac{\partial^{2}\left(\cdot \right)}{\partial x^{2}}+\lambda^{2}B_{11}\frac{\partial^{2}\left(\cdot \right)}{\partial y^{2}}\right),\\ L_{12}\left(•\right)=\nabla_{k}^{2}\left(\cdot \right)+\frac{\partial^{2}}{\partial x^{2}}\left(B_{10}\frac{\partial^{2}\left(\cdot \right)}{\partial y^{2}}+\lambda^{2}B_{11}\frac{\partial^{2}\left(\cdot \right)}{\partial x^{2}}\right)-\\ \;-2\frac{\partial^{2}}{\partial x\partial y}\left[\left(B_{10}-B_{11}^{}\right)\frac{\partial^{2}\left(\cdot \right)}{\partial x\partial y}\right]+\frac{\partial^{2}}{\partial y^{2}}\left(B_{11}^{}\lambda^{2}\frac{\partial^{2}\left(\cdot \right)}{\partial y^{2}}+B_{10}\frac{\partial^{2}\left(\cdot \right)}{\partial x^{2}}\right),\\ L_{21}\left(•\right)=\nabla_{k}^{2}\left(\cdot \right)+\frac{\partial^{2}}{\partial x^{2}}\left(B_{11}\lambda^{-2}\frac{\partial^{2}\left(\cdot \right)}{\partial x^{2}}+B_{10}\frac{\partial^{2}\left(\cdot \right)}{\partial y^{2}}\right)-\\ \;-2\frac{\partial^{2}}{\partial x\partial y}\left[\left(B_{10}-B_{11}^{}\right)\frac{\partial^{2}\left(\cdot \right)}{\partial x\partial y}\right]+\frac{\partial^{2}}{\partial y^{2}}\left(B_{10}^{}\lambda^{-2}\frac{\partial^{2}\left(\cdot \right)}{\partial x^{2}}+B_{11}\frac{\partial^{2}\left(\cdot \right)}{\partial y^{2}}\right),\\ L_{22}\left(•\right)=\frac{\partial^{2}}{\partial x^{2}}\left(A_{2}\frac{\partial^{2}\left(\cdot \right)}{\partial y^{2}}-\lambda^{-2}A_{1}\frac{\partial^{2}\left(\cdot \right)}{\partial x^{2}}\right)+\\ \;+\frac{\partial^{2}}{\partial y^{2}}\left(\lambda^{2}A_{1}\frac{\partial^{2}\left(\cdot \right)}{\partial y^{2}}+A_{2}\frac{\partial^{2}\left(\cdot \right)}{\partial x^{2}}\right)+2\frac{\partial^{2}}{\partial x\partial y}\left[\left(A_{1}^{}-A_{2}^{}\right)\frac{\partial^{2}\left(\cdot \right)}{\partial x\partial y}\right]. \end{array}$$

and:

(5) $$L\left(\left(•\right),\left(•\right)\right)=\frac{\partial^{2}\left(\cdot \right)}{\partial x^{2}}\frac{\partial^{2}\left(\cdot \right)}{\partial y^{2}}-2\frac{\partial^{2}\left(\cdot \right)}{\partial x\partial y}\frac{\partial^{2}\left(\cdot \right)}{\partial x\partial y}+\frac{\partial^{2}\left(\cdot \right)}{\partial y^{2}}\frac{\partial^{2}\left(\cdot \right)}{\partial x^{2}}$$

where *λ* stands for the length to width ratio.

The right hand sides of the governing equations with an account of transversal load and residual elastic–plastic deformations (with account of loading history) have the following form:

(6) $$Q_{1}=\lambda^{-1}\frac{\partial^{2}}{\partial x^{2}}\left(B_{10}T_{xx}^{0}+B_{11}T_{yy}^{0}\right)+2\frac{\partial^{2}}{\partial x\partial y}\left[\left(B_{10}-B_{11}\right)T_{xy}^{0}\right]+\lambda \frac{\partial^{2}}{\partial y^{2}}\left(B_{11}T_{xx}^{0}+B_{10}T_{yy}^{0}\right)-\;-\lambda^{-1}\frac{\partial^{2}T_{xx}^{1}}{\partial x^{2}}-2\frac{\partial^{2}T_{xy}^{1}}{\partial x\partial x}-\lambda \frac{\partial^{2}T_{yy}^{1}}{\partial y^{2}}+q,\;Q_{2}=\lambda^{-2}\frac{\partial^{2}}{\partial x^{2}}\left(A_{2}T_{xx}^{0}+A_{1}T_{yy}^{0}\right)+\lambda \frac{\partial^{2}}{\partial y^{2}}\left(A_{1}T_{xx}^{0}+A_{2}T_{yy}^{0}\right)-2\frac{\partial^{2}}{\partial x\partial y}\left[\left(A_{1}-A_{2}\right)T_{xy}^{0}\right],$$

stands for nonlinear operators, where, $T_{xx}^{0}$, $T_{yy}^{0}$, $T_{xx}^{1}$, $T_{xy}^{1}$, $T_{yy}^{1}$—contain temperature components and residual plastic deformations.

The relations between dimensional and nondimensional (bars) parameters are as follows:

(7) $$E=G_{0}\bar{E},\;G=G_{0}\bar{G},\;K=G_{0}\bar{K},\;q=G_{0}\frac{{\left(2h_{0}\right)}^{4}}{a^{2}b^{2}}\bar{q},\;T_{xx}^{0}=G_{0}\frac{{\left(2h_{0}\right)}^{3}}{ab}{\bar{T}}_{xx}^{0},\;F=G_{0}{\left(2h_{0}\right)}^{3}\bar{F},\;T_{yy}^{0}=G_{0}\frac{{\left(2h_{0}\right)}^{3}}{ab}{\bar{T}}_{yy}^{0},\;F=G_{0}{\left(2h_{0}\right)}^{3}\bar{F},\;T_{xx}^{1}=G_{0}\frac{{\left(2h_{0}\right)}^{3}}{ab}{\bar{T}}_{xx}^{1},\;\;T_{yy}^{1}=G_{0}\frac{{\left(2h_{0}\right)}^{3}}{ab}{\bar{T}}_{yy}^{1},\;K_{x}=\left(2h_{0}\right)a^{-2}{\bar{K}}_{x},\;K_{y}=\left(2h_{0}\right)b^{-2}{\bar{K}}_{y},\;B_{ik}^{}=G_{0}{\left(2h_{0}\right)}^{3}{\bar{B}}_{ik}^{},\;E_{ij}=G_{0}{\left(2h_{0}\right)}^{i+1}{\bar{E}}_{ij},\;(i=0,\ldots ,4,\;j=0,1),\;e_{r}={\left(\frac{2h_{0}}{a}\right)}^{2}{\bar{e}}_{r},\;e_{s}={\left(\frac{2h_{0}}{a}\right)}^{2}{\bar{e}}_{s},\;h=\bar{h}h_{0},\;A_{j}=\frac{1}{G_{0}(2h_{0})}{\bar{A}}_{j},\;(k=1,2),\;\lambda =\frac{a}{b},\;w=\left(2h_{0}\right)\bar{w},\;x=a\bar{x},\;y=b\bar{y}.$$

where: *G*_0_—the shear modulus characteristic value in the undeformed state; *G*—shear modulus; *K*—volumetric compression ratio; *h*_0_—shell thickness in its center; *K_x_*, *K_y_*—shell curvatures in *x* and *y* directions, respectively and *e_s_*—fluidity deformation.

Integrals with regard to shell thickness follow:

(8) $$E_{ij}=\int_{-h}^{h}\frac{Ez^{i}}{1+{\left(-1\right)}^{j}\nu }dz,\;T_{xx}^{j}=\int_{-h}^{h}\frac{Ez^{j}}{1-\nu^{2}}\left(\varepsilon_{xx}^{T}+\nu \varepsilon_{yy}^{T}\right)dz,\;T_{yy}^{j}=\int_{-h}^{h}\frac{Ez^{j}}{1-\nu^{2}}\left(\varepsilon_{yy}^{T}+\nu \varepsilon_{xx}^{T}\right)dz,\;T_{xy}^{j}=\int_{-h}^{h}\frac{Ez^{i}}{2\left(1+\nu \right)}\varepsilon_{xy}^{T}dz\;(i=0,\ldots ,4,\;j=0,1),$$

where $\varepsilon_{xx}^{T}=\alpha T+e_{xx}^{1p}$, $\varepsilon_{yy}^{T}=\alpha T+e_{yy}^{1p}$, $\varepsilon_{xy}^{T}=e_{xy}^{1p}\;$ stand for deformation depending on temperature and plastic deformation components of the deformation tensor in either relaxation ‘1P’ or at the time instant of the secondary loading (“2P”); as it was already mentioned that α is the coefficient of linear thermal expansion and *T* stands for the temperature function, whereas:

(9) $$\begin{array}{l} A_{j}=\frac{1}{2}\left(\frac{1}{E_{01}}+{\left(-1\right)}^{j+1}\frac{1}{E_{00}}\right),\;\\ B_{ik}=\frac{1}{2ih^{i-1}}\left(\frac{E_{i0}}{E_{01}}+{\left(-1\right)}^{k}\frac{E_{i0}}{E_{00}}\right),\;\left(i=1,3;\;j=1,2;\;k=0,1\right). \end{array}$$

The matrix $\left(\begin{array}{cc} L_{11} & L_{12}\\ L_{21} & L_{22} \end{array}\right)$ is symmetric, as in the case of an elastic problem (this observation is yielded by Betti’s theorem).

The governing Equations (3) are nondimensional (bars are omitted). It should be mentioned that PDEs (3) are obtained based on the kinematic Kirchhoff–Love model and the method of variable elasticity parameters [32]. The derivation of the governing equations is based on the theory of plasticity where *E* and *ν* are coupled with the shear modulus *G* and the bulk modulus *K* via the following relations:

(10) $$E=\frac{9KG}{3K+G},\;\nu =\frac{1}{2}\frac{3K-2G}{3K+G}$$

The following simplifications are introduced. We take *K = K*_0_ = *const* and owing to the hypothesized small elastic–plastic deformations, we have:

(11) $$G=\frac{1}{3}\frac{\sigma_{r}(e_{r})}{e_{r}},$$

where *σ*_r_ stands for the stress intensity, *e_r,_* describes the deformation intensity:

(12) $$e_{r}=\frac{\sqrt{2}}{3}{\left[{\left(e_{xx}-e_{yy}\right)}^{2}+{\left(e_{yy}-e_{zz}\right)}^{2}+{\left(e_{xx}-e_{zz}\right)}^{2}+1.5e_{xy}^{2}\right]}^{1/2},$$

and the following plane stress state formula holds:

(13) $$e_{zz}=-\frac{\nu }{1-\nu }\left(e_{xx}+e_{yy}\right),$$

where $e_{xx}$, $e_{yy}$, $e_{zz}$, $e_{xy}^{}$ are the strain tensor components for an arbitrary shell point.

It should be emphasized that *ν* is not constant.

## 3. Computational Method

In order to carry out the numeric computations, the dependence $\sigma_{r}(e_{r})$ can be defined in an arbitrary way. The system of nonlinear PDEs (3) is solved by the variational Bubnov–Galerkin method in higher approximations in the form proposed by Vlasov [33]. Namely, the system of approximating functions is assumed in the following form

(14) $$w=\sum_{c,b=1}^{N}P_{c,b}w_{c,b}(x,y),\;F=\sum_{c,b=1}^{N}R_{c,b}F_{c,b}(x,y).$$

where *w_c,b_* and *F_c,b_* stands for the system of approximating functions; *c*, *b* are the series terms number; *P_c,b_, R_c,b_* are constants to be determined from the Bubnov–Galerkin procedure.

In order to find the approximate value of the element *w* and *F*, we choose the coordinate systems of functions {*w_c,b_* (*x*,*y*), *F_c,b_* (*x*,*y*)} (*c, b* = 0, 1, 2,…) in (14), satisfying the following five requirements:

1. *w_c,b_* (*x*,*y*) ∈ *H_A_*, *F_c,b_* (*x*,*y*) ∈ *H_A_*, where *H_A_* is the Hilbert space, which we will call the energy space.
2. ∀ *c, b* functions *w_c,b_(x,y)* and *F_c,b_(x,y)* are linearly independent, continuous together with their partial derivatives up to the fourth order inclusive in the region Ω.
3. *w_c,b_ (x,y)* and *F_c,b_ (x,y)* satisfy the main boundary conditions (and the initial conditions, if any) exactly.
4. *w_c,b_ (x,y)* and *F_c,b_ (x,y)* have the completeness property in *H_A_*.
5. *w_c,b_ (x,y)* and *F_c,b_ (x,y)* must represent the *N* first elements of a function’s complete system.

We substitute (14) into (3) and after employment of the Bubnov–Galerkin procedure, the following system of nonlinear algebraic equations is obtained

(15) $$\left(\begin{array}{cc} a_{11} & a_{12}\\ a_{21} & a_{22} \end{array}\right)\cdot \left(\begin{array}{c} P\\ R \end{array}\right)+\left(\begin{array}{cc} a_{13} & 0\\ 0 & -a_{23} \end{array}\right)\cdot \left(\begin{array}{c} M\\ N \end{array}\right)+\left(\begin{array}{c} {\bar{Q}}_{1}\\ {\bar{Q}}_{2} \end{array}\right)=0,$$

where:

(16) $$P=\left(\begin{array}{c} P_{11}\\ P_{12}\\ \vdots \\ P_{1N}\\ \vdots \\ P_{NN} \end{array}\right),\;R=\left(\begin{array}{c} R_{11}\\ R_{12}\\ \vdots \\ R_{1N}\\ \vdots \\ R_{NN} \end{array}\right),\;M=\left(\begin{array}{c} M_{1111}\\ M_{1112}\\ \vdots \\ M_{111N}\\ \vdots \\ M_{NNNN} \end{array}\right),\;N=\left(\begin{array}{c} N_{1111}\\ N_{1112}\\ \vdots \\ N_{111N}\\ \vdots \\ N_{NNNN} \end{array}\right).$$

We substitute $\left(\sum_{c,b=1}^{N}P_{c,b}\right)\times \left(\sum_{k,l=1}^{N}R_{k,l}\right)$ and $\left(\sum_{c,b=1}^{N}P_{c,b}\right)\times \left(\sum_{k,l=1}^{N}P_{k,l}\right)$ by $M_{c,b,k,l}$ and $N_{c,b,k,l}$ respectively, where:

(17) $$a_{11}={\left(\int_{0}^{1}\int_{0}^{1}\left(L_{11}\left(w_{c,b}\right)\right)w_{f,v}dxdy\right)}_{f,v=1}^{f,v=N},\;a_{12}={\left(\int_{0}^{1}\int_{0}^{1}\left(L_{12}\left(F_{c,b}\right)\right)w_{f,v}dxdy\right)}_{f,v=1}^{f,v=N},\;a_{21}={\left(\int_{0}^{1}\int_{0}^{1}\left(L_{21}\left(F_{c,b}\right)\right)w_{f,v}dxdy\right)}_{f,v=1}^{f,v=N},\;a_{22}={\left(\int_{0}^{1}\int_{0}^{1}\left(L_{22}\left(F_{c,b}\right)\right)w_{f,v}dxdy\right)}_{f,v=1}^{f,v=N},\;a_{13}={\left(\int_{0}^{1}\int_{0}^{1}\left(L\left(w_{c,b},F_{c,b}\right)\right)w_{f,v}dxdy\right)}_{f,v=1}^{f,v=N},\;a_{23}={\left(\frac{1}{2}\int_{0}^{1}\int_{0}^{1}\left(L\left(w_{c,b},w_{c,b}\right)\right)F_{f,v}dxdy\right)}_{f,v=1}^{f,v=N},\;{\bar{Q}}_{1}={\left(\int_{0}^{1}\int_{0}^{1}Q_{1}w_{f,v}dxdy\right)}_{f,v=1}^{f,v=N},\;{\bar{Q}}_{2}={\left(\int_{0}^{1}\int_{0}^{1}Q_{2}F_{f,v}dxdy\right)}_{f,v=1}^{f,v=N}.$$

In order to solve the elastic–plastic problems through the Bubnov–Galerkin method (BGM), the program written in C++ is realized in the following way.

The following quantities are first introduced: geometric (*λ, K_x_, K_y_*) and physical (*K, E, ν, e_s_*) parameters, the analytical dependence *σ_r_* (*e_r_*) computational steps with regard to load Δ*q* or shell center deflection Δ*w*. Then the form of the approximating functions is defined depending on the introduced boundary conditions. In addition, we introduce the load type *q (x, y)*.

While employing the method of variable elasticity parameters, the computational process is realized in the following way. Having in hand the initial values of E, *ν*, *K*, formulas (8) and (9) yield the values of *E_ij_*, *A_j_*, *B_ik_* defined at the shell point (*x_n_*, *y_m_*). Then the system of Equation (15) is constructed. The Newton’s method yields the coefficients *P_ij_* and *R_ij_* with regard to Equation (14). The formulas:

(18) $$\begin{array}{l} e_{xx}=\varepsilon_{11}+z\chi_{11},\;e_{yy}=\varepsilon_{22}+z\chi_{22},\;e_{xy}=\varepsilon_{12}+z\chi_{12},\\ \varepsilon_{11}=A_{1}T_{1}+A_{2}T_{2}-B_{10}\chi_{11}-B_{11}\chi_{22}+A_{1}T_{xx}^{0}+A_{2}T_{yy}^{0},\\ \varepsilon_{22}=A_{2}T_{1}+A_{1}T_{2}-B_{10}\chi_{22}-B_{11}\chi_{11}+A_{2}T_{xx}^{0}+A_{1}T_{yy}^{0},\\ \varepsilon_{12}=2\left(A_{1}-A_{2}\right)S+\left(B_{10}-B_{11}\right)\chi_{12},\;\\ \chi_{11}=-\frac{\partial^{2}w}{\partial x^{2}},\;\chi_{22}=-\frac{\partial^{2}w}{\partial y^{2}},\;\chi_{12}=-2\frac{\partial^{2}w}{\partial x\partial y} \end{array}$$

allow to estimate deformations of the shell volume as well as the deformations intensity (relation (12)), and then Equations (10) and (11) yielded the shear modulus. In addition, the *E* and *ν* are numerically estimated at each point of the shell volume. However, as to be expected, the obtained values differ from the initial state. In order to avoid the problems of divergence, a new value of *ν* was substituted to Equation (12). Next, the improved value of *e_r_* is obtained, and new values of *E* and *ν* are defined. The mentioned internal process of iterations is repeated until the required accuracy is achieved and the obtained values of *E (x_n_,y_m_,z_p_*), *ν* (*x_n_,y_m_,z_p_*) are stored in the computer’s memory, where *x_n_, y_m_, z_p_* are the coordinates of the points set into which the shell volume is divided. Repeating the so far described computations for all *z_p_* the integration is carried out with regard to *z* and finally, the values of *E_ij_*, *A_j_*, B_ik_ as functions of *x*, *y* are defined.

However, the obtained values (usually) differ from the initial ones. They are taken again to carry out the computational process unless the given computational accuracy is achieved.

In order to solve the elastic–plastic problems of a considered shell we take the values of *E_ij_*, *A_j_*, *B_ik_* for linear elastic material as the first approximation. Then, the successive approximation of *E_ij_, A_j_*, *B_ik_* is achieved based on the values from the previous shell loading step. The latter choice of initial approximations allows for determination of *E* and *ν* by two to three iterations keeping the assumed accuracy of *ε* = 10^−5^.

## 4. Reliability of the Obtained Results

A solution of the system of nonlinear algebraic Equations (15) which are obtained by the Bubnov–Galerkin procedure are found with the help of the Newton’s iterative method. Integrations are carried out using the Newton–Cotes quadrature rules, where the shell volume is partitioned into 13 × 13 × 6 parts with regard to *x*, *y*, *z*, respectively.

Numeric differentiations are carried out with the help of finite-difference formulas. Owing to the worked out case studies, the shell volume partitions of 26 × 26 × 12 satisfy the required accuracy, and hence the obtained solution is reliable. In the case when the shell is supported by flexible non-stretched (nonelongated) ribs on its contour, and is subjected to uniformly distributed constant load, the following boundary conditions hold

(19) $$w=T_{xx}^{1}=T_{yy}^{0}=\varepsilon_{yy}=0,\;w=T_{yy}^{1}=T_{xx}^{0}=\varepsilon_{xx}=0,$$

where $T_{xx}^{1},T_{yy}^{1}$ stand for the moments and $T_{xx}^{0},T_{yy}^{0}$ for the forces defined at the shell center with regard to *x* and *y*.

The system of approximating functions satisfying the boundary conditions (19) take the following form

(20) $$w_{c,b}=F_{c,b}=\sin(c\pi x)\sin(b\pi y).$$

**Figure 2:**
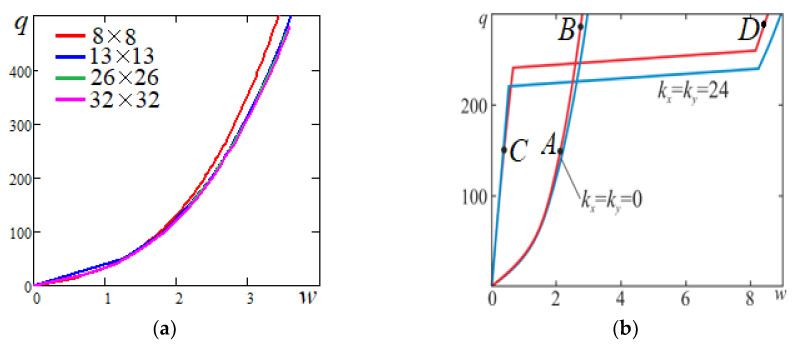
Dependence “load–deflection” (**a**) graphs comparison with different nodes number in the grid; (**b**) in the shell center obtained via the Bubnov–Galerkin method (BGM) in higher accuracy approximation (blue) and finite differences method (FDM) (red).

In order to check reliability of the used approach, we have compared the results obtained by the Bubnov–Galerkin method in higher approximations (*N* = 5) and by the finite differences method (FDM) of second order accuracy (*n* × *n* = 32 × 32). Figure 2a shows the load–deflection graph depending on the grid nodes number in the finite differences method of the second accuracy order for an elastic plate (8 × 8; 13 × 13; 26 × 26; 32 × 32). These results show that a 13 × 13 grid partition is sufficient to solve static problems. For an elastoplastic problem, a spatial grid of 13 × 13 × 6 is sufficient. Further studies were carried out with a partition of 13 × 13 × 6. We have fixed the shell curvatures *K_x_* = *K_y_* = 0, *K_x_* = *K_y_* = 24, and then the set up method was employed (see [34,35]). Table 1 presents the surfaces of deflection and stresses for the plate (*K_x_* = *K_y_* = 0) and the shell (*K_x_* = *K_y_* = 24) for the cases of pre-critical (*q =* 120) and postcritical (*q* = 300) loads. In the case of plate, the stress/deflection distribution character does not change qualitatively with regard to the load increase. In the case of the shell, the stress (Airy’s) function is positive (negative) for the pre-critical (postcritical) state, i.e., it changes its sign that serves as its stability criterion.

## 5. Algorithms for Account of the Residual Plastic Deformations

**Figure 3:**
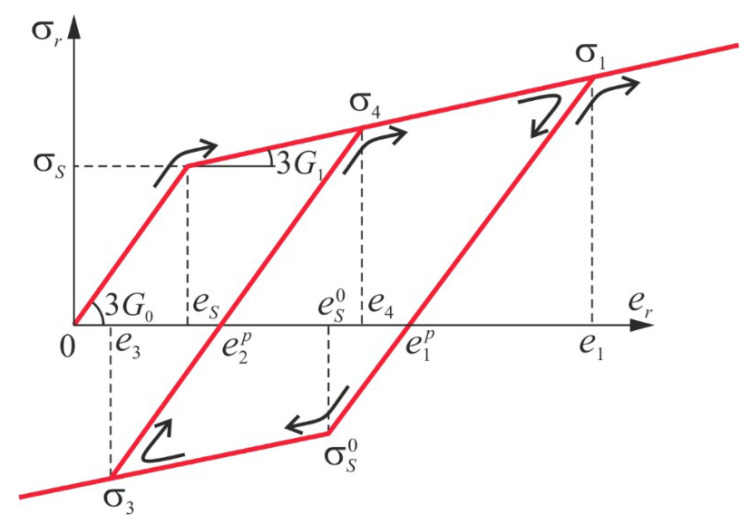
Dependence $\sigma_{r}(e_{r})$ constructed based on formulas (21) and (22).

We take the dependence *σ_r_* (*e_r_*) shown in Figure 3 and we trace a loading relaxation history of the shell element

(21) $$\sigma_{r}=3G_{0}e_{r}\;\;\mathrm{for}\;e_{r}<e_{s},\;\;\sigma_{r}=3G_{0}e_{r}+3G_{1}(e_{r}-e_{s})\;\;\mathrm{for}\;e_{r}\geq e_{s},\;$$

and:

(22) $$G=G_{0}\;\;\mathrm{for}\;e_{r}<e_{s},\;\;G=\frac{G_{0}e_{s}}{e_{r}}+G_{1}\left(1-\frac{e_{s}}{e_{r}}\right)\;\mathrm{for}\;e_{r}\geq e_{s},\;$$

In Figure 3 area (0; 0); (*e_s_*; *σ_s_*) describes elastic shell deformation, (*e_s_*; *σ_s_*); (*e*_1_; *σ*_1_) stand for the shell plastic deformation, unloading in the plastic deformation zone occurs in a straight line $\left(e_{1};\;\sigma_{1}\right)$; $\left(e_{s}^{0};\;\sigma_{s}^{0}\right)$ and secondary plastic deformations are denoted by $\left(e_{s}^{0};\;\sigma_{s}^{0}\right)$; $\left(e_{3};\;\sigma_{3}\right)$, whereas the repeated active loading is associated with notation $\left(e_{3};\;\sigma_{3}\right)$; $\left(e_{4};\;\sigma_{4}\right)$. The shell deformation process can be followed beginning from its counterpart nondeformed state and hence various variants of composition a computations of the quantities $e_{xx}^{1p},\;e_{yy}^{1p},\;e_{xy}^{1p},\;G$ can be numerically realized. We present and illustrate this possibility for an arbitrary chosen shell element.

Assume the considered shell element be under the active loading process where *de_r_* > 0. The shear modulus *G*, owing to the Mises criterion of plasticity, is equal to $G_{0}$ and the quantities $e_{xx}^{1p},\;e_{yy}^{1p},\;e_{xy}^{1p}$ are equal to zero. If $e_{r}\geq e_{s},$ the shear modulus is defined by the formula:

(23) $$G=G_{0}\frac{e_{s}}{e_{r}}+G_{1}\left(\frac{e_{r}-e_{s}}{e_{r}}\right).$$

The quantities $e_{xx}^{1p},\;e_{yy}^{1p},\;e_{xy}^{1p}$, $e_{1}^{p}$ get the following new values (on each stage of the loading process until *de_r_* > 0)

(24) $$\begin{array}{l} e_{xx}^{1p}=e_{xx}^{/}-\frac{1}{E}\left(\sigma_{xx}^{/}-\nu \sigma_{yy}^{/}\right),\\ e_{yy}^{1p}=e_{yy}^{/}-\frac{1}{E}\left(\sigma_{yy}^{/}-\nu \sigma_{xx}^{/}\right),\\ e_{xy}^{1p}=e_{xy}^{/}-\frac{2(1+\nu )}{E}\sigma_{xy}^{/},\\ \sigma_{r}=3G_{0}(e_{r}-e_{1}^{p}). \end{array}$$

The quantities with primes are defined in the beginning of the loading, whereas *E* and *ν* are computed using formulas (10) and they do not influence further computations (18). We need to recompute the quantities $e_{xx}^{1p},\;e_{yy}^{1p},\;e_{xy}^{1p},\;e_{1}^{p}$ at each stage of the active loading process, since the loading process may change its sign on at arbitrary loading stage. The latter requires knowledge of the last deformations $e_{xx}^{1p},\;e_{yy}^{1p},\;e_{xy}^{1p},$ and their intensity $e_{1}^{p}$on the previous loading stage, which in this case is known as the beginning of relaxation (it should be mentioned that an account of the loading and relaxation history is realized with accuracy up to the loading step).

In this relaxation case under the condition of lack of the plastic deformation, we have:

(25) $$de_{r}\leq 0,\;e_{r}<e_{1},\;G=G_{0}\frac{e_{r}-e_{1}^{p}}{e_{r}}$$

and the quantities $e_{xx}^{1p},\;e_{yy}^{1p},\;e_{xy}^{1p}$ are introduced into (18). If, after that the loading process is activated then unless $e_{k}<e_{1},$ the *G* modulus is defined by (25) and $e_{xx}^{1p},\;e_{yy}^{1p},\;e_{xy}^{1p}$ take part in the computations. However, if $e_{r}>e_{1},$ the modulus *G* is defined through (23) and the quantities $e_{xx}^{1p},\;e_{yy}^{1p},\;e_{xy}^{1p}$ and $e_{1}^{p}$ are compared to zero, i.e., they do not appear in (18) but they get new values. 

In the case of relaxation associated with the secondary plastic deformations, which corresponds to the fourth part of dependence *σ_r_* (*e_r_*) (see Figure 3), we have:

(26) $$\sigma_{r}=-3G_{0}+3G_{1}(e_{r}+e_{s}).$$

Here, we consider the material which obeys ideal Bauschinger’s effect with:

(27) $$G=-G_{0}\frac{e_{s}}{e_{r}}+G_{1}\frac{e_{r}+e_{s}}{e_{r}},\;de_{r}<0,\;e_{r}<e_{s}^{0}.$$

Here the quantities $e_{xx}^{1p},\;e_{yy}^{1p},\;e_{xy}^{1p}$ and $e_{1}^{p}$ obtained in the time instant of the relaxation beginning are taken into account in the further computations process. If, after that, we have *de_r_* > 0 (fifth part of the dependence *σ_r_* (*e_r_*)), then

(28) $$G=G_{0}\frac{e_{r}-e_{2}^{p}}{e_{r}},\;$$

and instead of the quantities $e_{xx}^{1p},\;e_{yy}^{1p},\;e_{xy}^{1p},\;e_{1}^{p},$ the quantities $e_{xx}^{2p},\;e_{yy}^{2p},\;e_{xy}^{2p},\;e_{2}^{p}$ are introduced. The latter are obtained on the last relaxation stage of the interval $e_{3}<e_{r}<e_{s}^{0}$ with regard to formulas (24) (here instead of values with one touch we use values with two touches found in a repeated load moment). However, the relaxation process can again change its sign (*de_r_* < 0) and if *e_r_ > e_3_* the modulus *G* is defined by formula (28) with inclusion of the quantities $e_{xx}^{2p},\;e_{yy}^{2p},\;e_{xy}^{2p},$ $e_{2}^{p}$.

If $e_{r}<e_{3},$ then *G* is defined by (27) and we take $e_{xx}^{2p},\;e_{yy}^{2p},\;e_{xy}^{2p},$ $e_{2}^{p}$ instead of $e_{xx}^{2p},\;e_{yy}^{2p},\;e_{xy}^{2p},\;e_{1}^{p}.$ Finally, if the secondary loading stage (fifth part) for *de_r_* > 0 is accompanied by the change of the plastic deformations, then for *e_r_ > e_4_* shear modulus *G* is governed by formula (23), whereas the quantities $e_{xx}^{1p},\;e_{yy}^{1p},\;e_{xy}^{1p},\;e_{1}^{p}$ are taken as equal to zero. Then, at each computational stage the quantities $e_{xx}^{3p},\;e_{yy}^{3p},\;e_{xy}^{3p},\;e_{3}^{p}$ are computed based on formulas (24), which opens the new cycle origin of the “load-relaxation” process.

In what follows we consider the neutron radiations of flexible rectangular shells stability. Lenskiy [4] pointed out that metal radiation through the fast neutrons flux implies change of their physical and metallic properties. It was observed that the largest radiation influence was exhibited by the magnitude of the carbon silicate material flow limit *σ_s_*. Radiation increase implies increase of *σ_s_*. In the steel F-212 V case, the functional and nonlinear dependence of *σ_s_* versus the total radiation flux is reported by Iliushin and Ogibalov [5].

If the radiation flux is perpendicular to material surface, then the dependence of the total versus amplitude can be approximated through the following formula [36]:

(29) $$N=N_{0}e^{2h(1\pm z)},\;-1\leq z\leq 1.$$

Assuming the following fixed parameters: 2h=2, $N_{0}=4\times 10^{19}n\nu t,$ ${\left(\frac{a}{2h_{0}}\right)}^{2}=1000,$ *G*_0_ = 0.8 × 10^6^ bar, *ν* = 0.3, *G*_1_ = 0 the plasticity flow limit is governed by the following formula:

(30) $$\sigma_{s}(z)=(5000\pm 1800z)$$

whereas the reported deformation intensity at the instant of the plasticity beginning is governed by the following formula:

(31) $$e_{s}=2.08\pm 0.75z.$$

Here the signs minus (−) and plus (+) refer to the shell radiation direction from its convexity and concavity, respectively. In the case of nonradiated material, we have *σ_s_* = 3375 bar, a *e_s_ =* 1.405.

Observe that in the latter case of the study, the radiation influence by the fast neutrons flux, the computational problem is reduced to computation of the plastic nonhomogenous (with regard to thickness) flexible shell made from an ideal elastic–plastic material. It should be mentioned that the worked out algorithm accepts arbitrary form of the dependence *σ_s_* (*z*), i.e., its either analytical or digital form.

## 6. Numeric Experiments and the Results Discussion.

In what follows we consider examples of the radiation direction influence on the shell behavior. The following geometric shell parameters are fixed: *K_x_ = K_y_* = 16, *K_x_ = K_y_* = 24.

The system of approximating functions to the computational solution is taken in the form of (20) and it satisfies the boundary conditions (19).

**Figure 4:**
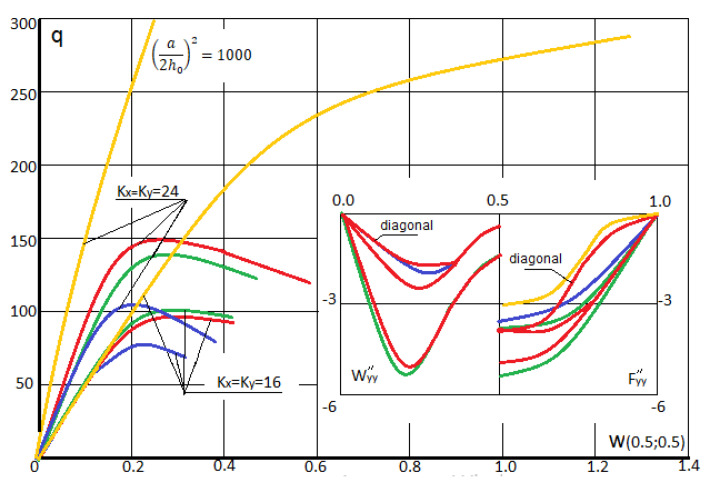
Load–deflection function in the shell center and shell cross-section I–I with regard to ${w^{\prime \prime }}_{yy}=\;\frac{\partial^{2}w}{\partial y^{2}}$ and ${F^{\prime \prime }}_{yy}=\;\frac{\partial^{2}F}{\partial y^{2}}$.

The computations are carried out in the way described earlier. We have taken *N* = 3 in (14), since further increase of the series terms quantity in (14) did not influence neither fundamental functions nor their second derivatives. The employed dependences *q (w)* are shown in Figure 4. They show the forms $w_{yy}^{//},$ $F_{yy}^{//}$ (second order derivatives) with regard to axial and diagonal lines for the values *w* = 0.2 and *w* = 0.4 in the shell center (upper and lower group of curves, respectively) for *K_x_ = K_y_* = 16 Red (green) curves correspond to the case then the shell radiation is employed from its convexity (concavity) side. In addition, blue color corresponds to the elastic–plastic solution, whereas yellow refers to the elastic solution.

**Figure 5:**
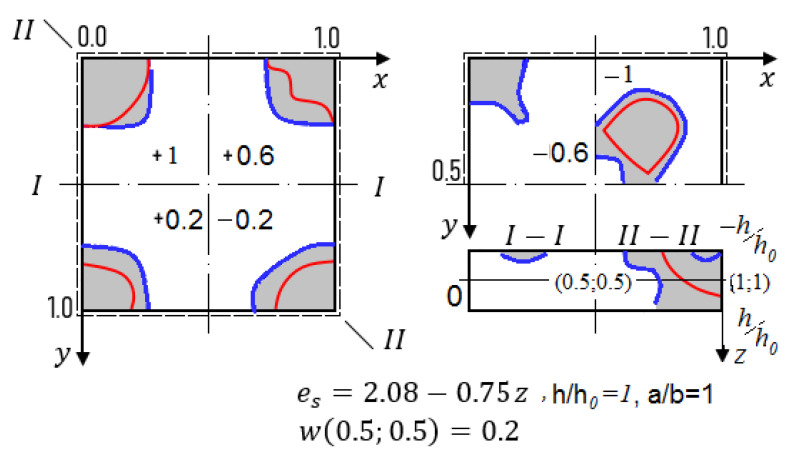
Views of the elastic–plastic deformation with regard to cross-section I–I and cross-section II–II along the shell thickness h = +1; +0.6; +0.2; −0.2; −0.6; −1 for *K_x_ = K_y_* = 16.

**Figure 6:**
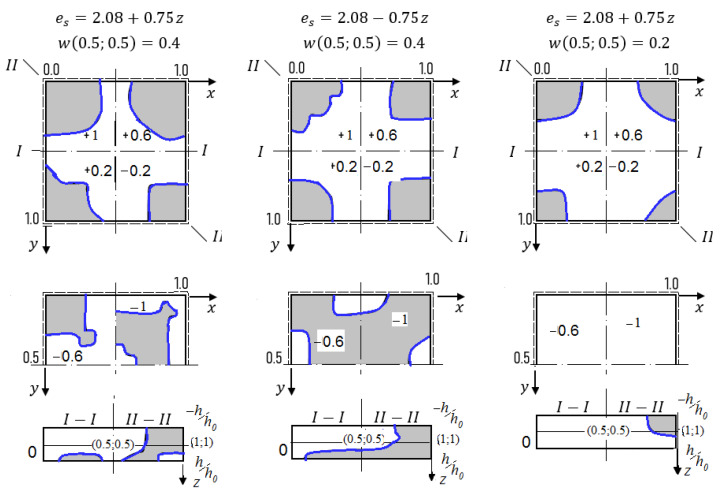
Views of the elastic–plastic deformation with regard to cross-section I–I and cross-section II–II along the shell thickness h = +1; +0.6; +0.2; −0.2; −0.6; −1 for *K_x_ = K_y_* = 24.

In the case of simply supported shells along their contour versus the geometric parameters *K_x_* and *K_y_* the radiation direction may influence the values of the upper critical loads (for instance, for *K_x_ = K_y_* = 24 the radiation employed from the convex shell side yields increase the upper critical load in amount of 47.5%, where from its concave side it achieves 35.2% or has the negligible influence). The similar consideration hold in the case for fixed *K_x_ = K_y_* = 16 the radiation employed from the convex shell side and from concave shell side yields increase of the upper critical load on amount of 34.6%. Distribution of the plastic zones for the plate with the fixed parameters *K_x_ = K_y_* = 16, *K_x_ = K_y_* = 24 along plane layers and along shell thickness for *w* = 0.2 and for *w* = 0.4 are reported in Figure 5 and Figure 6, respectively (blue curves correspond to plastic zones for the case of the radiation lack). 

## 7. Concluding Remarks

This work has been devoted to the mathematical modeling problem of flexible rectangular shallow shells stability under temperature field and subjected to nuclear radiations in order to improve technological and mechanical properties of the studied shells.

The developed theory and mathematical/numeric analyses allow estimating material strength and stability of structural members in two scale intervals, i.e., macro and micro scale. In the first case (macro) the engineering expected requirements refer to construction stability against the employed load as well as the mathematical exploitation longevity, whereas in the second case (micro) the critical role play sensitivity and stability of the construction working regimes under complexity of the input loading and thermal environment perturbations.

The carried out analysis based on the introduced theory and reliable computational algorithms allows formulating the following general conclusions of our research.

1. Theoretical background for analysis of the flexible nonhomogeneous shells under the radiation and temperature fields is given;
2. The method of solution to the derived nonlinear PDEs is based on the combination of the BGM in higher approximations, FDM and the Birger’s method with variable parameters;
3. We have found that radiation direction essentially influences both magnitude and the localization form of the plastic deformations—as well as the stresses magnitude in the shell middle surface;
4. The reported modeling and computational results allow to employ the radiation as a technological process improving the shell properties;
5. As a further study the following points are recommended for consideration:
  (a) construction of the mathematical nonlinear models with regard to vibrations of flexible elastic–plastic MEMS elements in a temperature field under external radiation exposure;
  (b) chaotic vibrations and transition scenarios from periodic to chaotic dynamics in the conditions of temperature and radiation fields;
  (c) development of mathematical models with an account of the relationship between the deformation and temperature fields subjected to radiation and temperature fields.
6. Experiments indicate that the mechanical properties of structural materials at certain dose of radiation (neutron fluence) are changed. These changes can be predicted by a proper and sophisticated mathematical modeling of structural members of constructions. Not accounting for the mentioned changes can lead either to excessive strength or to the appearance of additional radiation stresses and deformations, which can be economically inefficient and dangerous for structures.

## Figures and Tables

**Table 1:** Surfaces of deflection and stress of the plate and the shell.

$K_{x}=K_{y}=0$ (plate)			
q=150 (Point A (2, 150))		q=280 (Point B (2.8, 280))	
Deflection *w* (x, y)	Moment $\frac{M_{x}+M_{y}}{2}$	Deflection *w* (x, y)	Moment $\frac{M_{x}+M_{y}}{2}$
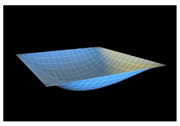	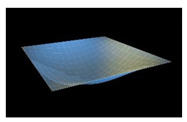	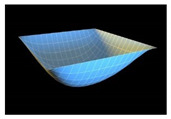	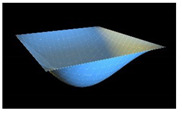
$K_{x}=K_{y}=24$ (shell)			
q=150 (Point C (0.5, 150))		q=280 (Point D (8.4, 280))	
Deflection *w* (*x*, *y*)	Average moment $\frac{M_{x}+M_{y}}{2}$	Deflection *w* (*x*, *y*)	Moment $\frac{M_{x}+M_{y}}{2}$